# Advances and Prospects in Antibacterial-Osteogenic Multifunctional Dental Implant Surface

**DOI:** 10.3389/fbioe.2022.921338

**Published:** 2022-05-24

**Authors:** Zixuan Wang, Baosheng Li, Qing Cai, Xiaoyu Li, Zhaoyi Yin, Birong Li, Zhen Li, Weiyan Meng

**Affiliations:** ^1^ Department of Dental Implantology, Hospital of Stomatology, Jilin University, Changchun, China; ^2^ Jilin Provincial Key Laboratory of Oral Biomedical Engineering, Changchun, China

**Keywords:** dental implant, surface modification, antibacterial, osteogenesis, osseointegration, soft tissue integration, immunity

## Abstract

In recent years, dental implantation has become the preferred protocol for restoring dentition defects. Being the direct contact between implant and bone interface, osseointegration is the basis for implant exerting physiological functions. Nevertheless, biological complications such as insufficient bone volume, poor osseointegration, and postoperative infection can lead to implant failure. Emerging antibacterial-osteogenic multifunctional implant surfaces were designed to make up for these shortcomings both during the stage of forming osseointegration and in the long term of supporting the superstructure. In this mini-review, we summarized the recent antibacterial-osteogenic modifications of the dental implant surface. The effects of these modifications on biological performance like soft tissue integration, bone osteogenesis, and immune response were discussed. In addition, the clinical findings and prospects of emerging antibacterial-osteogenic implant materials were also discussed.

## Introduction

With the continuous advancement of implant technology in recent years, dental implantation has become the preferred protocol for treating dentition defects. Professor Brånemark firstly proposed the osseointegration theory in 1966: the implant is in direct and close contact with the surrounding bone tissue under a light microscope, with no non-bone tissue intervention such as fibrous tissue in the middle (Guglielmotti et al., 2019). The osseointegration interface is a criterion of dental implant success, as well as the foundation for implant physiological performance. While intrinsic factors, surgical factors, local and systemic circumstances of the host, biomechanics of implant loading, implant design along with its upper support structure, and peri-implant hygiene conditions all affect osseointegration (Rupp et al., 2018). Moreover, the negative capability of surface forming osseointegration and post-operative infection have become critical factors that will be deleterious to osseointegration and influence the success of implantation, resulting in implant failure.

A wide range of methods has been proposed in basic experiments and clinical studies to improve the osseointegration of implants. Currently, methods to improve implant osseointegration include: changing local factors (such as radiation, oxide layer thickness, electric field, corrosion, etc.), physical therapy (vibration stimulation, etc.), drug therapy, implant surface modification, changing implant and abutment materials, but these methods also have the disadvantages of limited application and unsatisfactory results (Coelho et al., 2015) (Ota et al., 2016) (Guglielmotti et al., 2019) (Guglielmotti et al., 2019) (European Association for Osseointegration, 2019). For instance, the ultrasonic treatment also causes damage to the implant surface, which seriously affects the attachment of human osteoblast-like cells(Schwarz et al., 2003). Air-polished implant surfaces also did not exhibit a proliferation-promoting effect on osteoblasts (Matthes et al., 2017). The polished and plasma sprayed implant surfaces showed low alkaline phosphatase activity (Guizzardi et al., 2004), which affects the growth and metabolic activity of osteoblasts. Clinically applied coatings that promote osteogenesis, such as calcium phosphate coating, are less stable, have weaker adhesive strength with the substrate material, are prone to coating peeling and degradation, and are not conducive to forming long-term osseointegration (Schunemann et al., 2019).

Emerging antibacterial-osteogenic multifunctional implant surfaces were designed to make up for these shortcomings both during the stage of forming osseointegration and in the long term of supporting the superstructure. In this mini-review, we summarized the recent antibacterial-osteogenic modifications of the dental implant surface, and the effects of these modifications on the biological performance like soft tissue integration, bone osteogenesis, and immune response were discussed. In addition, this article discusses the clinical findings and prospects of emerging antimicrobial-osteogenic implant materials, providing a theoretical basis for improving implant osseointegration and promoting the long-term stability of implants.

## Antibacterial-Osteogenic Modifications of Dental Implant Surface

### Mechanical Treatment

The micro-nano multi-level microstructure on the implant surface refers to the implant surface topography formed by sandblasting, acid etching, polishing, and other processing technologies (Zhang et al., 2021), which determines the ability of early peri-implant bone formation and the level of bacterial adhesion (Souza et al., 2019). Nano-grooves, nano-pores, and other structures formed on the implant surface can promote the formation and mineralization of the extracellular matrix and accelerate the level of osseointegration (Mendonca et al., 2008; Souza et al., 2019). The implant surface’s nanomorphology also has a bactericidal function. The sharp edges of the nanostructure can stretch and rupture the bacterial cell wall, causing bacteria to lyse and reducing their viability (Jager et al., 2017). Zhu et al. used magnetron sputtering to create a tantalum-containing micro/nanocoating on titanium implants (Zhu et al., 2017). The coating not only improved the adhesion and spreading ability of rat bone marrow mesenchymal stem cells (rBMSCs), but also demonstrated antibacterial activity against *Streptococcus mutans* and *Porphyromonas gingivalis*. It has been revealed that the nanotextured surface has a more potent antimicrobial effect and can effectively induce osteogenic differentiation and calcium deposition (Kunrath et al., 2020). Wang et al. created a layered micro/nanomorphic polyetheretherketone implant with specific functional groups (amino and COOH/COOR) that displayed a good antibacterial activity. The micron/nanocoating and specific functional groups aided in the adhesion, proliferation, and osteogenic differentiation of MG-63 osteoblast-like cells (Wang et al., 2018) (Table 1).

**TABLE 1:** Antibacterial-osteogenic strategies of dental implant surface based on coating elements.

Coating classification	Material composition	Bacterial Strains	Osteogenesis	References
Biomacromolecular Coating	Vitamin E Phosphate	*S. aureus*	*In vivo*; promoting bone deposition and osseointegration	Lovati et al. (2018)
	TA/8DSS/PEG	*S. aureus*	BMSCs; promoting biomineralization and osseointegration	Han et al. (2021)
		*E. coli*		
	TA/HA/lysozyme	*S. aureus*	MC3T3-E1; promoting osteoblast mineralization and gene expression and ALP activity	Wang G. et al. (2021)
		*E. coli*		
	HA/HBD-3/BMP-2	*S. aureus*	hBMSC; promoting adhesion, proliferation, and osteogenic differentiation	Liu et al. (2018)
		*E. coli*		
	PDA/nZnO/CS/nHA	*S. aureus*	MC3T3-E1; promoting osteogenic differentiation and ALP expression	Wang Z. et al. (2021)
		*E. coli*		
Polymer Coating	Ti-RP/PCP/RSNO	*MRSA*	MC3T3-E1; upregulating the expression of ALP, Opn and Ocn	Li et al. (2020)
	SP@MX-TOB/GelMA	*S. aureus*	MC3T3-E1; improving the proliferation and diffusion of osteoblasts and the mineralization of calcium matrix	Yin et al. (2020)
		*E. coli*		
	SPEEK@SA(CGA)@BFP	*S. aureus*	MC3T3; improving cell adhesion and proliferation and osteogenic differentiation	He et al. (2019)
		*E. coli*		
	Ti-Br/PEG/RGD	*S. mutans, A. naeslundii*	MC3T3; promoting adhesion and proliferation of osteoblasts	Liu et al. (2016)
TiO2 Nanotube Coating	TNT/GelMA/PMAA-Cl/BMP-2	*S. aureus*	Osteoblasts; promoting cell adhesion, proliferation and differentiation	Jiao et al. (2020)
		*E. coli*		
	TNT/BMP2/(Chi/SL/Chi/Gel) _4_	*S. aureus*	Osteoblasts; improving cell viability, ALP activity, mineralization capacity and osteogenic gene expression	Sutrisno et al. (2018)
		*E. coli*		
	TNT/BMP2/LBLg	*S. aureus*	Osteoblasts; promoting differentiation of osteoblasts	Tao et al. (2019)
		*E. coli*		
	TNT/Au NPs/Pt NPs	*S. aureus*	hMSCs; enhancing osteogenic function	Moon et al. (2020)
Metal Ion/Nanoparticle Coating	Cu/Hier/Ti	*S. aureus*	Macrophages; creating a favorable inflammatory micro-environment for SaOS-2 cells, promoting osseointegration	Huang et al. (2019)
	AH-Sr-AgNPs	*S. aureus*	Macrophages; promoting macrophage polarization and differentiation of pro-osteoblasts	Li et al. (2019)
	PLGA/Ag/Fe_3_O_4_	*S.mutans*	Osteoblasts; promoting osteoblast proliferation	Yang et al. (2018)
	nAg/µCuO/PDA/SF	*S. aureus*	Ad-MSC; enhancing osteogenic differentiation	Yan et al. (2020)
		*E. coli*		

*ADA-Gen, alginate dialdehyde-gentamicin*; *A.naeslundii, Actinomyces naeslundii*; BFP, grafted peptide; BMP-2, Bone morphogenetic protein-2; CGA, chlorogenic acid; CS/Chi, chitosan; Cu-Hier-Ti, Cu-containing micro/nano-topographical bio-ceramic; *E.coli*, *Escherichia coli*; GelMA, gelatin methacrylate; HA, hydroxyapatite; HBD-3, human *β*-defensin 3; *LBLg, LBL/ADA-Gen; MRSA, Methicillin resistant Staphylococcus aureus*; MX, MXene; nAg, silver nanoparticles; nHA, nanocrystal hydroxyapatite; nZnO, ZnO nanoparticles; PCP, PVA/CS/PDA; PDA, polydopamine; PEG, polyethylene glycol; PLGA, poly(lactic-co-glycolic acid; PMAA-Cl, N-Cl modification poly (*N,N*′-methylene bis(acrylamide)); PVA, polyvinyl alcohol; RGD, arginine-glycine-aspartic; RSNO, a NO, donor of S-nitrosuccinic acid; SA, sodium alginate; *S.aureus, Staphylococcus aureus;* SL, sodium hyaluronate-lauric acid; *S.mutans, Streptococcus mutans*; SPEEK/SP, sulfonated polyetheretheretherketone; TA, tannic acid; TNT, TiO_2_ nanotubes; TOB, tobramycin; µCuO, copper oxide microspheres; 8DSS, 8 repeating units of aspartate-serine-serine.

UV photofunctionalization is the modification of titanium surfaces after UV treatment, which includes changes in physicochemical properties and enhanced biological capabilities (Ogawa, 2014). As a biologically inert material, titanium does not interact directly with cells and biomolecules. UV treatment can change the surface activity of titanium implants and transform the surface of titanium implants from hydrophobic to super hydrophilic. After UV irradiation, the electrostatic state of the titanium surface is changed, allowing direct adsorption of the desired cells. For example, one study found that UV treatment increased osteoblasts’ attachment, diffusion, proliferation, and mineralization on the titanium surface (Tsukimura et al., 2011). Compared to untreated implant surfaces, the bone morphology around UV-treated titanium implants changed significantly, allowing for rapid and stable osseointegration and promoting new bone formation with a near 100% bone-to-implant contact rate (Aita et al., 2009b; Kim et al., 2016). The UV-treated implant surface did not affect bacterial viability but significantly reduced bacterial adhesion and biofilm formation (De Avila et al., 2015). In addition, UV treatment effectively optimized the nanostructure of the titanium surface and promoted the adhesion and proliferation of osteoblasts (Tsukimura et al., 2011). In addition to promoting the proliferation of macrophages on the titanium surface and reducing the occurrence of inflammatory reactions (Lyu et al., 2019), UV treatment also increased the bioactivity of titanium dioxide nanotubes in human mesenchymal stem cells (Aita et al., 2009a). Thus, it is clear that UV treatment has positive implications for antimicrobial osteogenic modification of implant surfaces.

Bionanostructures have piqued the interest of researchers in recent years due to their excellent properties such as superhydrophobicity, self-cleaning, and antibacterial osteogenic dual-efficacy. Shahali et al. studied the nanostructures and properties of three cicadas and bionanostatically fabricated them using titanium nanopillars (Shahali et al., 2019). The bionanostructures were found to disrupt the morphology of *Pseudomonas aeruginosa* and *Staphylococcus aureus* (*S. aureus*), reduce bacterial adhesion, and promote osteoblast and actin adhesion and diffusion. In addition to cicada wings, surface nanostructures of dragonfly and butterfly wings, shark skin, gecko feet, taro and lotus leaves, and taro and lotus leaves have similar self-cleaning, bactericidal, and biocompatibility properties, making them more promising for implant surface modification (Jaggessar et al., 2017).

### Multifunctional Coating Biomacromolecular Coating

Biomacromolecular coating is the loading of biomacromolecules onto the implant surface through covalent bonding or layer-by-layer self-assembly, which has antibacterial properties and facilitates implant osseointegration (Cloutier et al., 2015). The various extracellular matrix proteins covering the implant surface, which affect cellular activity and trigger signaling pathways, play a crucial role in the interaction between the host and the implant (Liu et al., 2021).

Vitamin E phosphate implant coating reduced bacterial colonization of the implant surface. It increased bone deposition and osseointegration levels in an animal model of implant-associated infection by decreasing bacterial extracellular polysaccharide activity and immunomodulation (Lovati et al., 2018). Using the layer-by-layer self-assembly approach, implant surface was coated with tannic acid (TA), the biomineralization inducer 8DSS (8 repeat units of aspartate-serine-serine), and polyethylene glycol (PEG). PEG contributed to inhibiting bacterial adherence, 8DSS contributed to promoting biomineralization and osseointegration (Han et al., 2021). Wang et al. immobilized TA, hydroxyapatite (HA), and lysozyme on the implant surface, exhibiting a good antibacterial activity against *Escherichia coli* (*E. coli*) and *S. aureus*, meanwhile, the surface also promoted rapid adhesion and proliferation of mouse embryonic osteoblast precursor cells (Wang G. et al., 2021). In addition, Liu et al. developed a titanium implant coating containing nanohydroxyapatite, the natural antimicrobial peptide human *β*-defensin 3, and bone morphogenetic protein-2, which not only inhibited the growth of *S. aureus* and *E. coli*, but also promoted the adhesion, proliferation, and osteogenic differentiation of hBMSCs (Liu et al., 2018). Hence, the implant surface’s biomacromolecular coating had a substantial antibacterial and osteogenesis effect.

Chitosan (CS) comprises randomly arranged N-acetyl-glucosamine residues and glucosamine residues. By binding to negatively charged bacteria, CS can increase the permeability of bacterial cell membranes, thus acting as an antibacterial agent. Another antibacterial mechanism of CS is the interaction between its hydrophobic aryl substituents and the hydrophobic structure inside the bacterial cell wall. It has been found that the antimicrobial effect of CS is also influenced by its physical state and molecular weight, with longer alkyl substituents exhibiting stronger antimicrobial activity (Lu et al., 2016). CS coating can be deposited on the implant surface by electrophoretic deposition, sol-gel, dip-coating, spin-coating, and electrostatic spinning, along with osteogenesis-related factors, exercising dual antibacterial and osteogenic effects (Kumari et al., 2021). Ding et al. synthesized alkynyl-functionalized CS by reacting CS with 3-bromopropyne, showing better antibacterial activity against *E. coli* and *S. aureus* (Ding et al., 2013). Wang et al. created a composite coating (PDA/nZnO/CS/nHA) including polydopamine (PDA), zinc oxide nanoparticles (nZnO), and CS and nanocrystalline HA (nHA) (Wang Z. et al., 2021). Specifically, PDA doping reduced the porous titanium substrate’s surface roughness, wettability, and provided high adhesion to the deposited nZnO. While nZnO inhibited the growth of *S. aureus* and *E. coli*. Furtherly, the CS/nHA coating improved the osteogenic differentiation of MC3T3-E1 cells by up-regulating the expression of alkaline phosphatase. Hence, this multi-functional coating demonstrated a superior antibacterial osteogenic capacity.

### Polymer Coating

Grafting polymers on the implant surface, such as polyethylene glycol modifications, can inhibit bacteria’s ability to form biofilms and cause them to maintain a planktonic phenotype. Owing to the fact that most polymers lack antibacterial action, grafted polymers can only prevent bacterial colonization by preventing bacterial adhesion passively (Nejadnik et al., 2008). Hydrophilic polymer chains are usually physically adsorbed or covalently immobilized to the implant surface to prevent bacterial adhesion to the surface of the implant polymer coating, leaving the polymer layer well-hydrated, which is necessary for the development of antibacterial adhesion properties. A cross-linker can be generated between the polymer chains to make a hydrogel, allowing the polymer coating structure to retain more water without collapsing, improving the surface’s antibacterial adhesion capabilities (Swartjes et al., 2015).

Antimicrobial-osteogenic surfaces for hydrophilic implants have been studied using a variety of polymers such as polyethylene glycol, dextran, and hyaluronic acid (Liu et al., 2021). In order to achieve dual antimicrobial-osteogenic effects on the implant surface, it is possible to combine the antibacterial adhesion properties of the polymer with the bactericidal properties of the antimicrobial agent to improve the overall antimicrobial effect, in addition to grafting osteogenic-related factors on the surface of the implant polymer coating (Nejadnik et al., 2008). On the implant surface, block copolymers PF127 (Pluronic F-127) modified with antimicrobial peptide AMP, and PF127 modified with arginine-glycine-aspartate peptide RGD demonstrated good antibacterial adhesion, bactericidal, and tissue integration capabilities (Muszanska et al., 2014).

On red phosphorus nanomembranes of titanium implants, researchers created polyvinyl alcohol hydrophilic adhesive hydrogels (Ti-RP/PCP/RSNO) with CS, polydopamine, and NO-releasing donors. The structure could create NO with superoxide when exposed to 808 nm near-infrared light (NIR), which could upregulate the expression of Opn and Ocn genes as well as TNF-α, promoting osteogenic differentiation, and regulating inflammatory polarization while acting as an antibacterial agent (Li et al., 2020). Similarly, a novel multifunctional implant surface consisting of MXene nanosheets, gelatin methacrylate hydrogel, tobramycin, and bioinert sulfonated polyetheretherketone, showed strong antibacterial properties and osteogenic ability under 808 nm NIR illumination (Yin et al., 2020). Chlorogenic acid (CGA) and sodium alginate (SA) were grafted on the surface of sulfonated polyetheretheretherketone (SPEEK) implants to create the SPEEK@SA(CGA)@BFP hydrogel system. CGA was produced during the hydrogel’s disintegration to prevent bacterial growth. In contrast, osteoinductive growth factor (BFP) boosted osteoblast proliferation and differentiation. Hence the coating played a more significant part in antimicrobial bone regeneration (He et al., 2019). In addition, grafting polyethylene glycol-arginine-glycine-aspartic acid polymer brushes on the titanium surface effectively inhibited the growth of *Streptococcus mutans* and *Actinomyces naeslundii* and promoted osteoblast adhesion (Liu et al., 2016).

### TiO_2_ Nanotube Coating

Anodic oxidation, hydrothermal synthesis, and templating procedures can be used to form TiO_2_ nanotubes (TNT) on the surface of implants. TNT with nanoscale sizes increase their specific surface area, thus improving their photoelectrochemical characteristics (Hajjaji et al., 2018). TNT could promote the adhesion and proliferation of osteoblasts through osteogenic signaling pathways to achieve the long-term stability of implant osseointegration. For instance, TNT with nanomorphology provides locations for cell signaling *via* adsorbed proteins, which activate FAK and ERK1/2 pathways, enhancing hBMSCs cell motility, proliferation, and adhesion (Yang and Huang, 2019). In addition, TNT could achieve their dual antibacterial osteogenic function by forming micro-nano structures and combining them with metal ions, proteins, polymers, and medicines.

By disrupting bacterial cell walls and cell membranes, TNT can reduce bacterial adhesion and colonization, as well as kill bacteria by generating a pair of negatively charged free electrons and positively charged electron holes with strong redox properties through UV excitation. TNT react with water and oxygen to produce reactive oxygen species such as hydroxyl radicals, superoxide anions, and hydrogen peroxide (Liou and Chang, 2012). Drug molecules with antibacterial and osteogenic properties were loaded into TNT using various techniques, including physical adsorption, electrophoretic deposition, and magnetron sputtering. Changing the surface morphology of TNT can prolong the elution time of the drug and extend the duration of action of functional drug molecules against infection and contribute to bone regeneration (Wang K. et al., 2021). Jiao et al. prepared implant coatings containing BMP2 and GelMA/PMAA-Cl on TNT. They exhibited excellent antibacterial properties against the adhesion and growth of *S. aureus* and *E. coli* and played a positive role in osteoblasts’ adhesion, proliferation, and differentiation (Jiao et al., 2020).

In terms of enzyme response, Sutrisno et al. deposited bone morphogenetic protein 2 (BMP2) on the surface of titanium dioxide nanotubes to create a hyaluronidase-sensitive CS (Chi)/sodium hyaluronate-laurate (SL) coating (Sutrisno et al., 2018). Among them, hyaluronidase triggered the release of lauric acid from the SL coating and accelerated the release of BMP2 from the system. As a result, this coating not only inhibited *S. aureus* and *E. coli* from proliferation, but also boosted the expression of osteogenic markers such as collagen type I, osteocalcin, osteopontin, and alkaline phosphatase. In terms of pH response, a pH-responsive composite coating on TNT was developed that contains BMP2/alginate dialdehyde/gentamicin/CS (Tao et al., 2019). The release of gentamicin and BMP2 from the coating could be triggered by an acidic environment, which boasted antibacterial efficacy against *S. aureus* and promoted osteoblast development, alkaline phosphatase activity, mineralization ability, and the expression of osteogenic-related genes. In terms of photothermal response, Moon et al. created a nanoparticle coating on TNT that contained Au and platinum for the photothermal response (Moon et al., 2020). Under 470 nm visible light irradiation, the coating had strong antibacterial action against *S. aureus* and improved osteogenesis of human mesenchymal stem cells under 600 nm visible light irradiation.

### Metal Ion/Nanoparticle Coating

When a metal atom loses electrons, metal ions form as positively charged cations. Metal nanoparticles are small metal particles that range in size from 1 to 100 nm (Baranwal et al., 2018). Metal ions, in general, have a positive charge and are dissolved in water. Metal ions can use coulomb forces to firmly adsorb to bacterial cell membranes and react with bacteria for antibacterial purposes. Moreover, metal ions can be indirectly antibacterial *via* regulating macrophages and being direct contact bactericidal. Huang et al. created a copper-containing micro/nanomorphic bioceramic surface (Cu-Hier-Ti surface) that improved macrophages’ ability to take up and destroy bacteria, despite not being bactericidal. Cu^2+^ was carried to macrophage phagosomes by the copper transport signal protein ATP7A (Huang et al., 2019). It conducted a Fenton reaction with bacteria for sterilization and generated reactive oxygen species (ROS) in mitochondria to damage bacterial mitochondria. By modifying macrophages and increasing the expression of M1-type macrophage surface markers CD11c, growth factor BMP-6, OCN, and Runx-2, the Cu^2+^ surface promoted osseointegration. A dual delivery system (AH-Sr-AgNPs) on the titanium surface was created by alkali heat treatment (AH) for releasing Ag^+^ and Sr^2+^, which activated pro-osteoclast differentiation by regulating macrophage polarization and effectively resisted *S. aureus*-induced infections (Li et al., 2019).

Nanoparticles, on the other hand, are suspended in water. By interacting with bacterial cell membranes, cell walls, essential proteins, and enzymes, nanoparticles can operate as antibacterial agents, and their antibacterial efficacy is dependent on particle size (Friedman et al., 2013). Larger nanoparticles (>10 nm) release fewer ions, and their antibacterial capabilities are mostly manifested in direct contact with bacteria, whereas smaller nanoparticles (>10 nm) release more ions and their antimicrobial qualities are primarily manifested in direct contact with bacteria (Chernousova and Epple, 2013). As a result, modifying nanoparticles’ physicochemical attributes, such as size and shape, can increase their biological properties. Yang et al. developed a PLGA-encapsulated superparamagnetic Ag-Fe_3_O_4_ nanoparticle surface coating on implants with good antibacterial activity when exposed to a magnetic field to prevent *Streptococcus pyogenes* adherent colonization and promoted osteoblast proliferation and differentiation (Yang et al., 2018). Polyetheretherketone implants could be coated with copper CuO microspheres, silver nanoparticles, polydopamine, and filamentous protein. The coating released high doses of metal ions at pH 5.0, which killed 99.99% of planktonic bacteria, and low concentrations of metal ions in a physiological environment promoted ALP production, collagen secretion, calcium deposition, and NO production, thus promoting bone regeneration and osseointegration with being antibacterial contemporaneously (Yan et al., 2020).

## Effect of Antimicrobial-Osteogenic Modifications of Implant Surface on Soft Tissue Integration

The peri-implant soft tissue, being the biological barrier that protects the implant from bacterial invasion and maintains the long-term stability of the underlying bone tissue, is similar to natural gingival tissue and consists primarily of connecting epithelium and connective tissue (Chu et al., 2019). Whereas the absence of Sharpey fibers around the implant, combined with parallel collagen fibers encircling the implant surface, makes it easier for the epithelial layer to move towards the implant’s root side, disrupting the marginal closure (Ivanovski and Lee, 2018). As a result, implant surface modification should enhance epithelial and connective tissue to adhere to the implant surface.

The implant’s rigid properties and two-dimensional surface make it difficult for soft tissues to integrate optimally with the implant. Developing a “buffer zone” between the titanium implant and the soft tissue can attract cell migration and infiltration, restore the cellular microenvironment, and improve tissue integration (Leng et al., 2021). A hybrid hydrogel coating with ZnO nanoflowers and methacrylated gelatin and methacrylated hyaluronic acid was created on the titanium surface (Leng et al., 2021). This coating acted as a buffer for inward cell development and soft tissue integration, promoting fibroblast growth and CTGF and COL-I expression while inhibiting *S. aureus*-induced infections. Similarly, Mathur et al. created a bionanofiber coating doped with silver nanoparticles and electrospun gelatin(Mathur et al., 2021). They found that this coating had an excellent antibacterial activity against *S. aureus* and *E. coli* and promoted fibroblast adhesion, growth, and differentiation.

Recently, Matter et al. developed a triple-functional implant surface, which possessed antimicrobial, osteogenic, and soft tissue integration properties (Matter et al., 2021). Bioactive glass, cerium dioxide nanoparticles, and 2% of zinc doping were used to create the intriguing nanocoating. The coating not only enhanced the integration of bone and soft tissues by stimulating the growth and adhesion of osteoblasts and fibroblasts, but also prevented the growth of methicillin-resistant *S. aureus*.

## Effect of Antimicrobial-Osteogenic Modifications of Implant Surface on Bone Regeneration

While resisting bacterial infection, the antimicrobial-osteogenic modification on the implant surface should promote the adhesion, proliferation, osteogenic differentiation of bone marrow mesenchymal stem cells, and subsequently increase their mineralization capacity, therefore upregulating the expression of osteogenic markers as type I collagen, osteocalcin, osteopontin, and ALP.

However, previous studies have also shown that some antimicrobial modifications on the implant surface can inhibit osteogenesis. For example, zinc ions could disrupt cellular energy metabolic processes by generating ROS to kill bacteria. Nevertheless, once larger doses of zinc ions were incorporated into Ca-Si-based bioactive glass ceramics, the excess zinc ions affected the deposition of calcium ions and affected the formation and growth of hydroxyapatite (Wu et al., 2008). A similar study showed that zinc ions promoted the death of osteoblasts and facilitated the process of bone resorption during the bacteriocidal process (Hu et al., 2012). ZnO nanorods, which had the antimicrobial effect, also reduced the cell viability of macrophages and decreased the adhesion and proliferation of macrophages (Zaveri et al., 2010). Therefore, the balance of antimicrobial and osteogenic effects of zinc ions is crucial for the effective antimicrobial-osteogenic modifications of implant surfaces. Lactoferrin is known for its ability to bind iron, which will lead to the discovery of its antibacterial activity. However, it has been found that lactoferrin could decrease the proliferative activity of osteoblasts and the bone formation capacity due to the conformational changes of lactoferrin (Wang et al., 2013). Hence, given the differences in their activities of antibiosis and osteogenesis, the molecules for implant surface modification should be considered comprehensively to achieve antimicrobial-osteogenic dual function.

Nowadays, lactoferrin has been successfully loaded on the implant surface, which effectively inhibited the adhesion and proliferation of *Streptococcus sanguis* and *S. aureus* and promoted osteogenic differentiation (Chen et al., 2021). Ding et al. loaded poly-l-glutamic acid and polyallylamine hydrochloride, silver nanoparticles, mesoporous silica nanoparticles, and polydopamine on a titanium surface to address the previously mentioned problem(Ding et al., 2020). The coating inhibited the growth of *Streptococcus. aureus* and increased the thickness of bone trabeculae and the volume and area of new bone. Yuan et al. also created a functional molybdenum disulfide (MoS_2_)/polydopamine -arginine-glycine-aspartate coating on the surface of titanium implants that not only resisted bacterial infection of *Streptococcus. aureus* and *Escherichia. coli* when illuminated with NIR but also increased the expression of osteogenesis-related genes (Yuan et al., 2019). Thereupon, the inhibitory effect of active antimicrobial molecules on osteogenesis should not be ignored in the research phase of antimicrobial-osteogenic modifications on implant surfaces.

## Effect of Antimicrobial-Osteogenic Modifications of Implant Surface on Immunological Aspects

**FIGURE 1:**
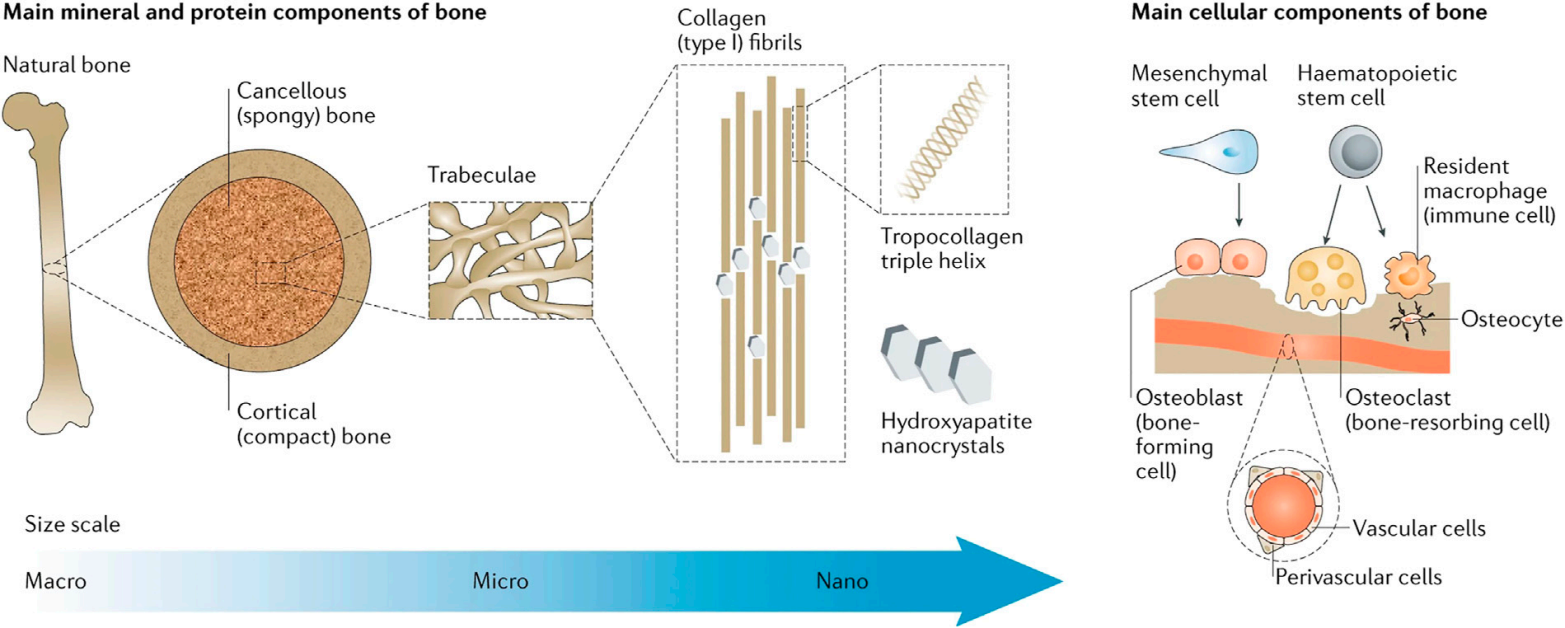
Bone physiology for biomimicry of candidate biomaterials for bone-tissue engineering. Reproduced with permission from Koons et al. (2020).

After implant implantation, macrophages play a crucial part in the immune cascade response (Koons et al., 2020) (Figure 1). Pattern recognition receptors (PRRs) on the cell surface of the body, such as macrophages, recognize bacteria and their metabolites as pathogen-associated molecular patterns (PAMPs) in the early stages of bacterial infection. TNF-*α*, IL-1, IL-1*β*, IL-6, and NO are produced by macrophages that have been activated and polarized to the M1 type, a pro-inflammatory phenotype that produces a great variety of pro-inflammatory mediators. Pro-inflammatory factors have vasodilatory and chemotactic actions, which might attract more leukocytes to the site and speed up PAMP clearance (Liu et al., 2014). The first inflammatory response after biomaterial implantation aids tissue repair and regeneration; however, the overabundance of M1-type macrophages can lead to chronic inflammation, stymie wound healing, and damaged tissue repair (Batool et al., 2021). M2 macrophages, which have immunosuppressive and anti-inflammatory properties, are essential in this situation.

Researchers tried to alter the physical properties of the implant surface to a more osteointegration-friendly M2-macrophage type that facilitate angiogenesis and bone regeneration around implants, namely immunomodulating the implant surface and modulating the macrophage phenotype (Kim, 2020). Nanoscale surface treatment of implant surfaces can influence macrophage morphology and phenotypic characteristics, while functionalized bioactive molecular materials can increase anti-inflammatory factors and decrease the release of pro-inflammatory factors by adjusting their surface hydrophilicity, surface charge, and surface coating (Batool et al., 2021).

In recent years, polyarginine coatings became prevailing for their excellent properties. In addition to its superb surface antimicrobial properties, polyarginine has been associated with the phenotypic transformation of macrophages and the synthesis of factors related to vascular endothelial cells. Researchers have developed multifunctional implant coatings containing gelatin, aldehyde-modified hyaluronic acid, and polyarginine, which effectively resisted infections caused by *S. aureus* and promote anti-inflammatory polarization and angiogenesis (Knopf-Marques et al., 2019). Other scholars designed implant coatings based on polyarginine, hyaluronic acid, and natural host defense peptide (catestatin), which were found to have long-term stable antimicrobial activity, and reduce the pro-inflammatory potential of macrophages, decrease the release of chronic inflammation-related factors, and facilitate tissue remodeling and healing (Ozcelik et al., 2015). In addition, polyarginine with a degree of polymerization of 30 reduced lipopolysaccharide-stimulated macrophage inflammatory response, accelerated fibroblast migration in macrophage/fibroblast co-culture systems and had a positive effect on wound healing (Gribova et al., 2022).

TNT have been found to reduce macrophage inflammatory responses by inhibiting MAPK and NF-κB pathways and reducing the expression levels of mitogen-activated protein kinase signaling molecules p38, ERK1/2, and JNK phosphorylation, which promotes macrophage polarization to M2 type to promote tissue repair (Neacsu et al., 2015).

Metal ions, such as Ag^+^ and Sr^2+^, can modify macrophage polarization toward the M2 phenotype and enhance pro-osteoblast development while reducing *S. aureus* survival in the dual delivery system (AH-Sr-AgNPs) on titanium surfaces (Li et al., 2019). Silver nanoparticles have also been found to efficiently suppress inflammation by modifying TLR-mediated signaling and decreasing TLR ligand-mediated IL-6 production. It can also be used to reduce reactive oxygen species levels and restrict T-lymphocyte proliferation, thus reducing IL-2 release and controlling the immune system’s inflammatory response (Ninan et al., 2020).

Different from the above research, Huang et al. polarized macrophages to the M1 phenotype purposely by creating a Cu-containing micro/nanomorphic bioceramic surface, which activated Cu translocation signaling in macrophages through the regulation of integrin (α5, αM, β1, β2) and TLR (TLR-3, TLR-4, Myd88, and Ticam-1/2) signaling to exert some inflammatory effects. The surface inhibited *Streptococcus. aureus* growth and proliferation, and intriguingly increased the proliferation and differentiation of human osteoblasts SaOS-2 (Huang et al., 2019). Comprehensively, polarizing to M2 phenotype of macrophages is not the unique protocol for promoting osteointegration. Appropriate activation of M1 phenotype of macrophage might be instrumental in solving inflammation more rapidly.

## Perspective and Challenges

The osseointegrated interface is the hallmark of successful dental implants and the basis for the physiological function of the implant. Since plaque biofilm is closely related to peri-implantitis, inhibiting microbial adhesion and biofilm formation on the implant surface and promoting the formation of stable osseointegration are the main strategies to prevent and treat peri-implantitis. Currently, methods to improve the antimicrobial properties of implants include creating antimicrobial implant surface morphology, forming an antimicrobial coating. However, most of these methods have disadvantages, such as limited application and unsatisfactory results. Owing to the complex environment inside the oral cavity, antimicrobial implant surface modification should be optimized to a multifunctional modification to promote soft and bone tissue bonding while effectively inhibiting bacteria. The targeting, responsiveness, and stability of the antimicrobial-osteogenic coating, and long-term stability of the surface-tissue interface are also issues that need to be investigated in the future. Improving the binding of the active ingredients and whether the active ingredients can be released on demand with long-term efficacy need to be thoroughly explored.
